# Has the Sudden Health Emergency Impacted Public Awareness? Survey-Based Evidence from China

**DOI:** 10.3390/bs12020021

**Published:** 2022-01-25

**Authors:** Xiaojia Guo, Jingzhong Li, Fang Su, Xingpeng Chen, Yeqing Cheng, Bing Xue

**Affiliations:** 1 College of Geographical Science, Shanxi Normal University, Taiyuan 030031, China; guoxj@sxnu.edu.cn; 2 College of Urban Planning and Architecture, Xuchang University, Xuchang 461000, China; lijingzhong@xcu.edu.cn; 3 School of Economics and Management, Shaanxi University of Science & Technology, Xi’an 710021, China; sufang@sust.edu.cn; 4 College of Earth and Environmental Sciences, Lanzhou University, Lanzhou 730000, China; 5 College of Geography and Environmental Sciences, Hainan Normal University, Haikou 571158, China; yqcheng@iga.ac.cn; 6 Institute of Applied Ecology, Chinese Academy of Sciences, Shenyang 110016, China; xuebing@iae.ac.cn

**Keywords:** human-land relationship, sudden public health emergencies, COVID-19 pandemic, environmental cognition, humanistic characteristics, geographical differences

## Abstract

Public environmental cognition is an important basis for optimizing environmental management and reducing tensions between humans and land. Although the level of environmental cognition is a gradual process under normal conditions, it often changes qualitatively because of major public emergencies. During the 2019 new coronavirus epidemic (COVID-19), the most significant public health event in recent years, 24,188 national samples were obtained based on a network survey. The comprehensive evaluation method was used to assess the impact of major public events on public environmental cognition and the characteristics of spatial and temporal distribution. The findings are as follows. (1) During the epidemic period, sudden public health emergencies effectively promoted the national residents’ environmental awareness, whether urban residents or rural; most respondents generally agreed with the concept of “respect nature and cherish life”. (2) The environmental cognition of national residents was higher in the northwest and lower in the northeast of China, which is suitable for economic and social development and humanistic tradition. (3) There was a clear positive correlation between environmental awareness and education level. (4) During the epidemic, nervousness of respondents had a negative effect on environmental cognition. This study provides scientific support and a basis for decision making for the government to carry out environmental management optimization and improve the ecological and environmental cognition of the public, as well as devise effective intervention mechanisms with different time and space dimensions for similar future public health emergencies.

## 1. Introduction

“Environmental cognition” refers to the cognitive feedback formed by a human’s integration of environmental information and continuous accumulation [1], which is important to understand the internal connection between human behavior and environment [2,3]. Thus far, COVID-19 has caused more than 300 million infections and over 5 million deaths, resulting in substantial impacts on people’s lives as well as posing a significant threat to achieving the United Nations’ 2030 Sustainable Development Goals (SDGs) on schedule [4]. As an important basic support for sustainable development, environmental awareness has been influential and inspirational in the studies of environment protection and green development, not only in the developed Western world but also in the developing regions of the world [5,6,7]. It is clear that people with good environmental awareness can better understand and support the government’s decisions and actions in response to climate change [8,9,10], thus, enhancing public environmental awareness to promote sustainable development has become an important part of regional development policy with a global scope [11,12,13].

Environmental awareness is closely related to education, income, and other internal personal conditions [5,14,15,16]; continuous imperceptible influence of daily education and management from families, schools, and society is a common way to increase it [17,18,19]. Meanwhile, external conditions that change dramatically, especially a sudden public event stimulus, result in immediate public environmental awareness change. Examples of these changes include oil pollution in the Gulf of Mexico [20], water pollution in the Songhua River [21] and the Chernobyl and Fukushima nuclear accidents [22,23]. So far, existing research has shown that public environmental health events can also raise public environmental awareness, such as with Ebola [24,25] and Middle East Respiratory Syndrome [26]: these deadly infections have attracted more attention to environmental factors and prompted interval circulation management. The research results of an international teamwork from the United States, the United Kingdom, and Australia clearly show that COVID-19 is not a laboratory construct or a purposefully manipulated virus, but the product of natural evolution [27].

No doubt, COVID-19 is a sudden public health emergency characteristic of being the fastest spreading and most difficult to control and prevent infection since the establishment of the People‘s Republic of China on 1 October 1949 [28], so much so that the central government has clearly stated its intention “to mobilize the public and carry out special environmental health education to guide the masses in good hygienic habits, advocate for civilized health and environmentally friendly daily practices” [29]. Hence, we sought to answer several necessary questions. Does a sudden public health emergency, such as the COVID-19 pandemic, effectively promote an environmental awareness? Does this influence have different characteristics in different spaces, across China covering 2463 county-level administrative units in 31 provincial-level administrative regions (excluding Hong Kong, Macao, and Taiwan)? Are there significant correlations between the selected social characteristics, such as education level, different occupational types and age, and environmental awareness? This paper attempts to find prompt and reasonable answers to these three questions to further understand the impact of the relationship between major public events and public environmental cognition level, and to better promote environmental education and management post-epidemic.

However, most of the current research focuses on the pathologic studies of SARS, MERS, and COVID-19 [27,30,31], and the research on public cognition usually focuses on the psychological stress response to the epidemic and daily protective behaviors [32,33,34], while few scholars carry out relevant research on the impact of infectious diseases from the perspective of environmental cognition. In addition, research has failed to carry out targeted research on the regional characteristics and public group characteristics during public health emergencies, which could not have reflected the environmental cognition differences among different regions, thus ignoring the individual differences in the face of sudden disasters. Therefore, this study analyzes the change trend of environment cognition in the process of the COVID-19 outbreak and reveals the human factors and socioeconomic and spatial distribution characteristics of the individual cognitive environment. As a result of the past long-term urban and rural dual system, other studies have found that urban and rural residents may have cognitive biases and geographical differences regarding environmental awareness and behavior; however, we found that the environment cognition of respondents has improved in both areas. Regardless of land and class, wealth, race, age, or education, the threat and infection of a virus is the same for everyone. In conclusion, this study provides scientific support and a reference for decision-making to enable the government to prevent and tackle major public health emergencies events as well as carry out environmental protection promotion and education, so as to enhance public ecological and environmental literacy.

## 2. Materials and Methods

Towards the end of January 2020, due to the widespread diffusion of COVID-19, Wuhan declared a lockdown (at 10 a.m. on 23 January 2020). Successively, the entire country launched the most comprehensive, strict, and thorough prevention and control measures. Although the epidemic situation was different in various provinces, all residents faced the same quarantine policy.

China has released information on COVID-19 in a timely, accurate, open, and transparent manner. People have immediate access to real-time, dynamic information related to the outbreak and the movement path of those infected. In addition, apps such as WeChat and Alipay can be used to effectively reduce the level of panic and spread of epidemic-related rumors. Internet users’ awareness of a certain event or thing on the Internet is highly expressed by the frequency of Internet users’ search index; the higher the search frequency, the higher the netizens’ attention to a certain event. When the epidemic was raging, it was essential to perform orderly, coordinated, timely, and comprehensive research. Hence, in order to analyze the changing trend of environmental cognition in the course of the COVID-19 outbreak, a questionnaire was designed in two parts and included 11 questions. Part one contains the basic demographic information of the interviewee, mainly including age, gender, address of residence during the epidemic, education level, occupation type, and so on. Part two is the core of the questionnaire and includes four questions about the cognitive change of the natural environment and the fluctuation of the interviewees’ emotional state from 21 January to 18 February 2020, shown as follows:

Q1. Do you feel anxious and depressed about COVID-19? Answer: (A: Yes); (B: No).

Q2. What is your current view on “human and nature should live in harmony and respect nature” compared to before the epidemic? Answer: (A: disagree); (B: no feeling); (C: slightly agree); (D: strongly agree).

Q3. What has been your emotional state from 20 January to 17 February (4 weeks):

(1) Week 1 (20–27 January) Answer: (A: calm); (B: slightly worried); (C: nervous and afraid); (D: strong fear); (E: numb);
(2) Week 2 (27 January–3 February) Answer: (A: calm); (B: slightly worried); (C: nervous and afraid); (D: strong fear); (E: numb);
(3) Week 3 (3–10 February) Answer: (A: calm); (B: slightly worried); (C: nervous and afraid); (D: strong fear); (E: numb);
(4) Week 4 (10–17 February) Answer: (A: calm); (B: slightly worried); (C: nervous and afraid); (D: strong fear); (E: numb).

Q4. What are the main reasons for your psychological state (multiple choice; choose no more than 3 answers)? Answer: (A: local people who are susceptible); (B: susceptible occupation); (C: rumors); (D: high fatality rate); (E: high infection rate); (F: shortage of medical resources); (G: other).

From 21 January to 28 February 2020, an online questionnaire was conducted based on the snowball sampling method to investigate the emotional status and environmental cognition of residents during the epidemic blockade and at-home quarantine being enforced, covering 2463 county-level administrative units (municipal districts or county-level cities) in 31 provincial-level administrative regions (excluding Hong Kong, Macao and Taiwan). A total of 24,215 samples were collected, of which 24,188 were valid samples, with a response rate of 99.89%. On the whole, there were 14,493 female respondents in the valid questionnaire accounting for 59.9%, and the group aged 20–49 accounted for 76.2%. Among the respondents, there were 21,819 respondents with college degrees or above, accounting for 90.2%. Correspondingly, in terms of occupation, college students accounted for 53.1%, followed by enterprise and institution employees who accounted for 27.7%, which is consistent with the education level of respondents. One-third of respondents lived in urban centers, and one-third lived in rural areas, which is essentially consistent with China’s urban–rural structure. The nuclear family with 3–4 members accounted for as much as 60%, which is consistent with China’s “one child” basic national policy since July 1971; as a result, the family size has been shrinking, with the average family size dropping from 4.41 in 1982 to 3.17 in 2006 [35]. Taking the COVID-19 outbreak events as the background, respondents self-compared to the views on “respect nature and build a harmonious relationship between human and nature” on behalf of environmental cognition level. Here, changes of respondents’ environmental awareness from ‘without’ COVID-19 to ‘with’ the pandemic can be divided into four levels: “slightly disagree, no change, slightly agree, or strongly agree”. Accordingly, the levels scored from 1 to 4 points; the higher the score, the greater the improvement of environmental perception during the home quarantine period due to the outbreak of the epidemic.

The change in the environmental cognition characterization was based on each interviewee’s own self-comparison (before epidemic and after the outbreak). In order to further analyze the relationship between environmental cognition and physical quality, cultural literacy, professional experience, and psychological state during the epidemic, we selected the age, gender, education level, occupation type, and psychological state as independent variables which passed the significance level test, and environmental cognition as dependent variables, and adopted the multiple logistic model for empirical analysis. The formula is as follows:

(1) $$status_{i}=education_{i}+gender_{i}+occupation_{i}+age_{i}+mood_{i}+\varepsilon_{i}$$

Here, subscript *i* represents the *i*th sample, *ε* is a random perturbation term. Dependent variable *status_i_* is a measure of environmental cognition for the *i*th sample. The independent variables include the *education*, *gender*, *occupation*, *age* and *mood*. The variables in the model are explained in Table 1.

## 3. Results

**Figure 1:**
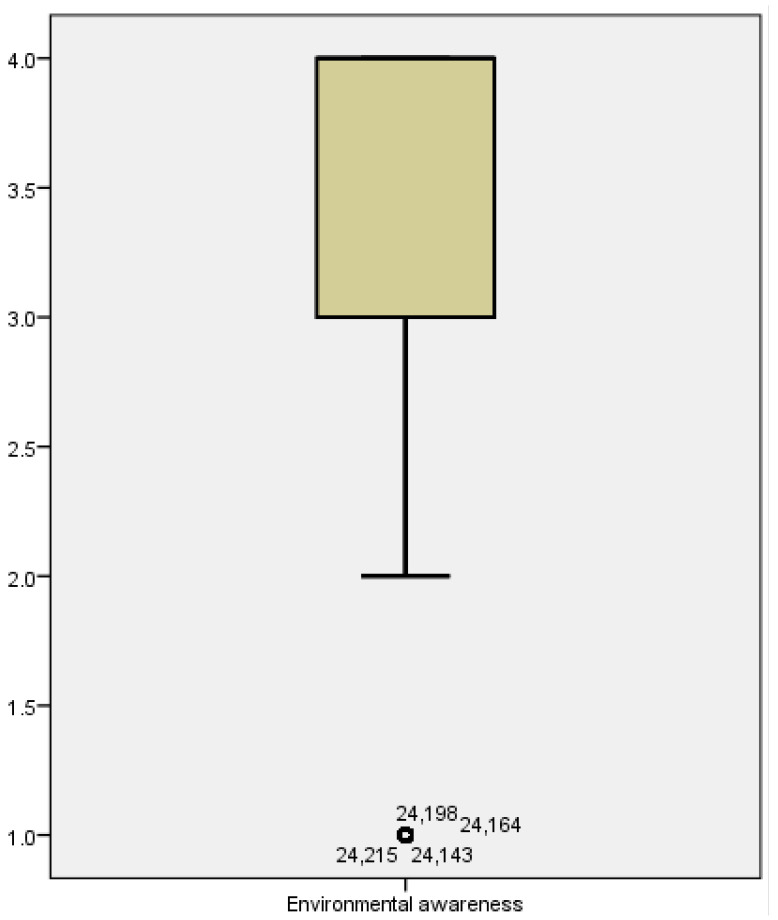
Boxplot of environmental awareness.

Figure 1 shows that the mean value of public environmental cognition level is 3.307, the mode is 4, and the overall environmental cognition presents a positive skew distribution. Regardless of land, class, wealth, race, age, or education, the threat and infection of a virus is the same to everyone, hence the public’s feedback is immediate and consistent.

**Figure 2:**
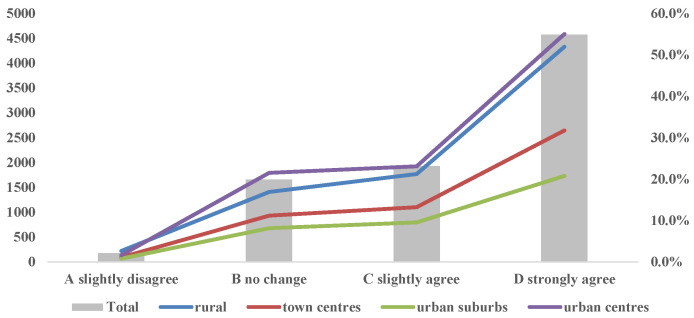
Interviewees’ views on “human and nature should live in harmony and fear nature”.

In particular, 78 percent of respondents felt more strongly about the idea that people should live in harmony with nature and respect nature, while only 2.1 percent agreed less with this compared with prior to the pandemic. To a certain extent, it indicates that after the occurrence of COVID-19, people’s environmental awareness level of being in awe of nature and respecting nature significantly improved, and this event promoted the positive improvement of public environmental protection (see Figure 2).

Moreover, the investigation also found that whether respondents lived in a city, town, or the countryside, their environmental cognition largely improved to a large extent, with only a few getting worse.

**Figure 3:**
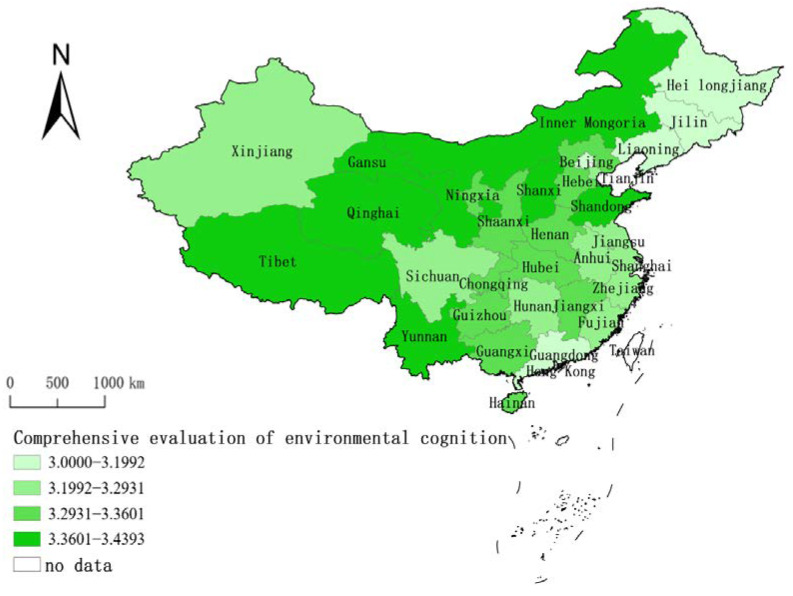
Spatial distribution characteristics of comprehensive environmental cognition.

Based on the calculation and analysis of the entire sample, the public environmental cognition of 31 provinces and regions (excluding Hong Kong, Macao, and Taiwan) in China was evaluated at the provincial level, and its spatial distribution characteristics were described (see Figure 3). The results show that there have been significant positive changes in respondents’ environmental cognition throughout China (the average score of all provinces was greater than 3), but there are also relative spatial differences, and Shanxi had the highest level in the positive direction of public environmental cognition with 3.439 average value. Based on the natural discontinuity method, environmental cognition was divided into four levels. The first level included six provinces and cities: Shanghai, Beijing, Jilin, Guangdong, Liaoning, and Heilongjiang. The second level included eight provinces and cities: Zhejiang, Fujian, Xinjiang, Tianjin, Jiangsu, Sichuan, Anhui, and Hunan. The third level included 10 provinces and cities: Jiangxi, Guangxi, Henan, Hubei, Shaanxi, Chongqing, Guizhou, Hainan, Ningxia, and Hebei. The fourth level included seven provinces: Inner Mongolia, Qinghai, Gansu, Tibet, Yunnan, Shandong, and Shanxi. On the whole, the spatial distribution pattern of environmental cognition was low in the northeast and high in the northwest.

Source: The raw base map from the website of the Ministry of Natural Resources of China (http://bzdt.ch.mnr.gov.cn/, accessed on 30 March 2020) was plotted by ArcGIS 10.2 software (ESRI^®^), and its Drawing Review Number is GS (2016) 2893.

**Figure 4:**
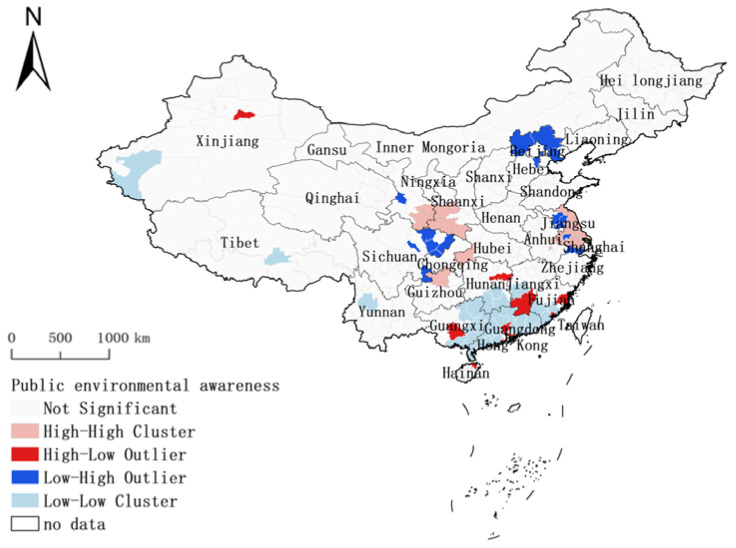
The spatial agglomeration of public environmental cognition.

Based on the geospatial correlation method, the spatial agglomeration of the degree of public environmental cognition friendliness was studied (see Figure 4). According to the research results, the global Moran’s I index was 0.0316, the sample variance was 0.0002, Z score was 2.7998, and *p* value was 0.005, indicating that the probability of the agglomeration phenomenon of public environmental cognition in geographical space was less than 1%, and it mainly presented the random distribution pattern of patch Mosaic. Among them, Guangdong region was a “low–low” cluster; Jiangsu province and the border region of Shaanxi–Gansu province was a “high–high” cluster, that is, Guangdong region was the cluster with low environmental cognition friendliness; Jiangsu and Guanzhong Plain was the cluster with high environmental cognition friendliness. In terms of environmental cognition spatial agglomeration, the differentiation of “high–high” and “low–low” indicates that there was obvious dependence and heterogeneity in the local spatial distribution of public environmental cognition in China during the epidemic period.

There was a positive spatial spillover effect of environmental cognition in the border region of Jiangsu province and Shaanxi–Gansu province, while there was a negative spatial spillover effect of environmental cognition in Guangdong province. Meanwhile, there was no spatial spillover effect between Hubei province and its neighboring provinces, and there was no “high–high” or “low–low” agglomeration of environmental cognition, indicating that the impact of the COVID-19 epidemic on residents’ environmental cognition does not have a neighborhood effect on the whole.

The survey shows that the environmental cognition of interviewees is positively correlated with their education level; that is, environmental cognition is more likely with the improvement of education level. However, with the improvement of educational background, the standard error, skewness, and kurtosis decrease continuously, indicating that the environmental cognition of highly educated groups is more consistent and the degree of dispersion is less so (see Table 2). The average environmental cognition of the group with college degree and above was found to be high, and minimize the error, skewness, and kurtosis, which suggests that generally, the highly educated group had higher environmental awareness through an environmental protection course in school. This finding is also consistent with the conclusion of other scholars, such as Li, who found significant differences in the environmental behavior of people with different education levels, and the residents’ environmental protection behavior scores to be highly positively related to level of education, who along with having higher culture awareness, were more likely to take practical action to protect the ecological environment [36].

In Table 3, the result of the correlation analysis with public environmental cognition shows that the order from smallest to largest is “gender” < “education” < “age” < “place of residence” < “occupation” < “mood”, with values of 0.138, 0.049, 0.031, −0.013, −0.015, and −0.071, respectively. Age, gender, and education level have a positive impact on public environmental cognition, and significant at the confidence level of 5%. In contrast, occupational type and mental state during the epidemic had negative effects on public environmental cognition, and were significant at 10% and 5% confidence levels, respectively. The correlation between environmental cognition and settlement was not significant, most likely due to the fact that regardless of where residents lived, they were all required to stay home.

Furthermore, through choosing age, gender, education level, occupation type, and mental state that passed the significance level test as independent variables, and taking environmental cognition as a dependent variable, multiple logistic regression was conducted in order to analyze the relationship between public, environmental cognition with an individual humanistic, geographical factor during the epidemic.

Based on the likelihood ratio test, the significance level P of the chi-square value of the multinomial logistic model was 0.000, indicating that the model was well-fitted and the simulation results were reliable (see Table 4).

In Table 4, we can see that the influence on the public environmental cognition shows a gradual downward trend, and even positive effects can be seen to change to negative effects with increasing age. The order from smallest to largest was below 20 (0.255), 20–29 (0.094), 30–39 (0.001), 40–49 (−0.132), 50–59 (−0.175), etc. However, the influence of each age group on the environmental cognition was not significant. The odds ratio of the “under 20 years old” group was 1.290, that is, the probability of people’s environmental awareness in the better level was 1.290 times than before COVID-19. Moreover, the odds ratio decreased gradually with the increase of age, indicating that the probability of the “below 20” in the better grade of environmental cognition was higher than that of other age groups. To a great extent, this shows that compared with other age groups, the environmental awareness of the “below 20” group is the easiest to improve for environmental cognition. This is closely related to environmental education in the school system, and substantial research has placed emphasis on environmental education for young people [18].

As shown in Table 4, occupational types have positive effects on environmental cognition. The order from smallest to largest was entrepreneur (0.000), middle-level cadres (0.112), student (0.134), governmental employee (0.182), peasant (0.200), retiree (0.269), etc. Among them, the regression coefficient of the “governmental employee” group was 0.182 and the odds ratio was 1.199, which was significant at the confidence level of 5%. In other words, the environmental awareness of employees working in national enterprises and government was 1.199 times more likely to be in a higher level than other occupation type groups. This is closely related to the result of Table 3 that the environmental cognition of students is also positive at 10% significant confidence level.

Obviously, gender and psychological status had significant effects on environmental cognition, both at the 1% confidence level (see Table 4). Gender has a positive effect on environmental cognition, indicating that gender shows significant differences in environmental cognition under the stimulus of an emergency. This is probably because women are more sensitive than men and more likely to relate to nature. The nervousness of the interviewees under the epidemic situation has had a negative impact on environmental cognition, especially for the women and elders. The surveys also showed that 35% of the females were terrified, while 33% of the males felt terrified. Moreover, the respondents over 60 years of age who felt anxious accounted for 37.6%, while only 29.8% of respondents under 30 years of age felt depressed. Lack of key information and insufficient knowledge of environmental protection led to the spread of public nervousness, to a certain extent, and this reflects that the improvement of environmental cognition can properly alleviate residents’ negative emotions towards the epidemic situation.

## 4. Discussion

The human-land relationship is the mutual influence and feedback between humans and the natural environment [37], in which the human is the key factor and plays a core role in influencing the ecological environment [38]. The study has revealed that once the external conditions change dramatically, this will lead to an immediate change in public environmental cognition. Usually, a sudden public health emergency event has a positive impact, which is consistent with the research findings of Joseph et al., (2014) and MD Oh et al., (2018) [24,26]. As a result of the past long-term urban and rural dual system, settlements have formed a different composition and life concept of the natural human landscape in urban and rural areas [39]. Correspondingly, this can also create cognitive biases and geographical differences between urban and rural residents’ environmental awareness and environmental behavior [35,40,41]. However, our investigation found that regardless of whether people lived in a city, town, or the countryside, the environmental cognition of the main respondents improved, while it worsened in only a few.

However, different groups have different environmental perceptions and responses to epidemics. As far as gender was concerned, 35% of females reported to be terrified, which was two percentage points higher than that of males. In short, women were more likely to feel anxious and depressed than men as well as feel prone to conflict and tension with their families; this is consistent with the research results of Cheng and Qing [42,43]. The survey showed that the environmental cognition of interviewees was positively correlated with their education level, and this is also consistent with the conclusion of other scholars, such as Li, who found people with higher culture inhabitants were more likely to take practical action to protect the ecological environment [36]. Huang et al. found that residents’ awareness of public participation in environmental assessment in Beijing is significantly positively correlated with their educational level, especially the proportion with college or above level education, reaching as high as 76.78% [44]. The survey showed that with the increase of age, the public’s environmental awareness gradually decreased. This is probably because knowledge reserve and memory decreases over time; therefore, it is especially necessary to strengthen environmental protection education for middle-aged and elderly populations who are already out of school education. Correspondingly, regarding recognition of the environment and epidemic situation, the respondents over 60 years reporting feeling anxious accounted for 37.6%, while for those under 30, only 29.8% felt depressed. This is probably because older people are more likely to be infected than young people, consistent with other research [45,46].

At the same time, the research is mainly based on network random sampling, lack of information in the farmer, elderly, retiree, and junior high school groups, while the highly educated group, government staff, and student number was too large, leading to the conclusion of extrapolation by certain restrictions. Nevertheless, we believe our findings provide meaningful results with useful implications for policy makers and reference for social study. This is most likely because during that time, the whole country was kept in rigorous quarantine and people could only stay at home, which meant the face-to-face interview was not possible. In the future, more detailed work will be conducted with a questionnaire survey carried out both online and offline and covering a more diverse range of people.

## 5. Conclusions

In the context of the COVID-19 epidemic, the study found that public awareness of environmental protection tended to be positive. Regardless of rural and urban regions, most interviewees’ awareness of environmental protection improved. On the whole, people’s environmental cognition showed a spatial distribution pattern of low in the northeast and high in the northwest. Under the impact of the epidemic, the public’s awareness of environmental protection in respect of nature has been greatly enhanced. There is a positive correlation between the public’s environmental awareness and education level. Different occupational types show different cognitive levels. It is worth noting that family and community education and communication are the most extensive and important, but also the most easily ignored, and a harmonious and stable family and community are undoubtedly the premise of winning over the epidemic. It is necessary that government and community management pay more attention to women, the elderly, and single-parent families, those who are more likely to feel anxious and depressed, those who are prone to conflict and tension with their families, and those who are more prone to be infected.

When optimizing environment management and reducing tensions between humans and land, it is imperative to improve citizens’ environmental hygiene literacy in response to public health events, pay attention to environmental education in schools and public welfare publicity of environmental protection, and carry out in-depth, extensive, and lasting environmental protection work. Governments should vigorously carry out publicity and popularization of health knowledge, such as public safety and disease prevention and control, and guide residents to firmly establish their concept of being responsible for their own personal health.

## Figures and Tables

**Table 1:** Variable setting and meaning in the multiple logistic model.

Variables	Variable Definition
Status	Environmental cognition (1 = slightly disagree, 2 = no change, 3 = slightly agree, 4 = strongly agree)
Education	Level of education (1 = primary school and below, 2 = junior high school, 3 = high school or secondary school, 4 = college and above)
Gender	Gender (1 = male, 0 = female)
Age	Age (1 = below 20, 2 = 20–29, 3 = 30–39, 4 = 40–49, 5 = 50–59, 6 = 60 and above)
Occupation	Occupational types (1 = employees of enterprises and institutions, 2 = middle level and above leading cadres, 3 = entrepreneurs, 4 = students, others)
Mood	Are you anxious or depressed about the severe form of the epidemic? (1 = Yes, 0 = No)

**Table 2:** Relationship between education level and environmental cognition.

Education Level	Statistics	Environmental Cognitive Score	Standard Error
primary school and below	Mean	3.050	0.085
	Skewness	−0.575	0.199
	Kurtosis	−1.052	0.396
junior high school	Mean	3.110	0.036
	Skewness	−0.525	0.092
	Kurtosis	−1.111	0.184
high school or secondary school	Mean	3.260	0.023
	Skewness	−0.725	0.063
	Kurtosis	−0.868	0.126
college and above	Mean	3.320	0.006
	Skewness	−0.854	0.017
	Kurtosis	−0.550	0.033

**Table 3:** Correlation analysis with public environmental cognition.

Item	Age	Gender	Education	Occupation	Settlements	Mental State
Pearson correlation	0.031 **	0.138 **	0.049 **	−0.015 *	−0.013	−0.071 **
Significance (two tails)	0.000	0.000	0.000	0.018	0.050	0.000
N	24,215	24,215	24,215	24,215	24,215	24,215

Note: * = 10% and ** = 5% indicate significant level of 10% and 5%, respectively.

**Table 4:** Simulation results of public environmental cognition.

Item	Regression Coefficient	Clustering Robust Standard Error	Wald Test Value	Odds Ratios EXP (B)
age = below 20	0.255	0.238	1.147	1.290
age = 20–29	0.094	0.234	0.160	1.098
age = 30–39	0.001	0.233	0.000	1.001
age = 40–49	−0.132	0.233	0.322	0.876
age = 50–59	−0.175	0.230	0.578	0.840
gender	0.382 ***	0.033	130.838	1.465
education = primary and below	0.039	0.242	0.026	1.040
education = junior middle school	0.128	0.117	1.192	1.137
occupation = governmental employee	0.182 **	0.071	6.458	1.199
occupation = middle-level cadres	0.112	0.095	1.389	1.119
occupation = entrepreneur	0.000	0.122	0.000	1.000
occupation = student	0.134 *	0.076	3.132	1.143
occupation = peasant	0.200	0.151	1.757	1.222
occupation = retiree	0.269	0.205	1.720	1.308
mental state	−0.145 ***	0.034	17.923	0.865

Note: * = 10%, ** = 5%, and *** = 1% indicate significant level of 10%, 5%, and 1%, respectively.

## Data Availability

Not applicable.
